# The neonatal assessment manual score (NAME) for improving the clinical management of infants: a perspective validity study

**DOI:** 10.1186/s13052-021-01012-9

**Published:** 2021-03-07

**Authors:** Andrea Manzotti, Marco Chiera, Matteo Galli, Erica Lombardi, Simona La Rocca, Pamela Biasi, Jorge Esteves, Gianluca Lista, Francesco Cerritelli

**Affiliations:** 1 RAISE lab, Foundation COME Collaboration, Corso Europa 29 - 66054 Vasto (Italy), Pescara, Italy; 2 Division of Neonatology, “V. Buzzi” Children’s Hospital, ASST-FBF-Sacco, Milan, Italy; 3 Research Department, SOMA, Istituto Osteopatia Milano, Milan, Italy

**Keywords:** Neonatology, Manual assessment, Haptic perception, Body volume, Autonomic nervous system, Prematurity, Touch, NICU

## Abstract

**Background and objectives:**

The Neonatal Assessment Manual scorE (NAME) was developed to assist in the clinical management of infants in the neonatal ward by assessing their body’s compliance and homogeneity. The present study begins its validation process.

**Methods:**

An expert panel of neonatal intensive care unit (NICU) professionals investigated the NAME face and content validity. Content validity was assessed through the content validity index (CVI). Construct validity was assessed using data collected from 50 newborns hospitalized in the NICU of “Vittore Buzzi” Children Hospital of Milan, Italy. Kendall’s τ and ordinal logistic regressions were used to evaluate the correlation between the NAME scores and infants’ gestational age, birth weight, post-menstrual age, weight at the time of assessment, and a complexity index related to organic complications.

**Results:**

The CVIs for compliance, homogeneity, and the whole scale were respectively 1, 0.9, and 0.95. Construct validity analysis showed significant positive correlations between the NAME and infants’ weight and age, and a negative correlation between the NAME and the complexity index (τ = − 0.31 [95% IC: − 0.47, − 0.12], *p* = 0.016 and OR = 0.56 [95% IC: 0.32, 0.94], *p* = 0.034 for categorical NAME; τ = − 0.32 [95% IC: − 0.48, − 0.14], *p* = 0.005 for numerical NAME).

**Conclusions:**

The NAME was well accepted by NICU professionals in this study and it demonstrates good construct validity in discriminating the infant’s general condition. Future studies are needed to test the NAME reliability and predictive capacity.

## Introduction

A valid and reliable diagnostic instrument is the foundation of clinical practice since it gives accurate information about the patient’s health and clinical conditions [1]. Validity is an essential property since assessing validity answers the question of whether the instrument measures exactly what it proposes to measure [2].

In the neonatal intensive care unit (NICU), taking care of infants is complicated due to the comorbidities associated with maternal stress [3], labor and delivery complications [4, 5], and preterm births [6]. Infants can show several pathologies that threaten their survival [6], and many routine-care procedures can be both stressful and painful for the infants and, hence, influence their neurological development in the short- and long-term [7, 8]. Besides, NICUs are growing in complexity and professionals need an ever more throughout knowledge about infants’ physio-pathology [9–11].

Therefore, all neonatologists and NICU professionals need reliable and valid diagnostic tools that provide measurements about the infant’s development, growth, and clinical conditions to manage the infant optimally [12].

In the NICU babies are handled hundreds of times per day [13]. Despite this, touch remains largely non-specific and effects on infants understudied [8]. Moreover, many commonly used clinical assessment procedures rarely use touch as a diagnostic tool [12, 14, 15]. Therefore, it would be beneficial to define and develop a structured touch-based approach to assess infants.

However, assessing the validity of manual approaches has always been complicated: a previous attempt to define an evaluative procedure for the preterm newborns [16] showed several problems indeed. One limitation was the manual nature of the procedure, which made it difficult to extend it to other NICU professionals. Another limitation was the lack of standardized manual procedures in neonatology: the procedures were somehow adapted from the ones on adults even though the anatomy of newborns, especially if preterm, and adults are different [16]. Besides, typical weaknesses of manual approaches are the lack of reliability among different operators and the sensitivity of the procedures themselves, which can be strongly biased by the operator’s subjective experience [17].

The Neonatal Assessment Manual scorE (NAME) model – a new assessment procedure in the neonatology field – was developed for overcoming these difficulties [18]. The NAME aims to evaluate the infant’s general clinical conditions through the assessment of how the body tissues respond and adapt to manual stimulation, i.e., static light touch. It is designed to produce a score that could correlate with the infant’s clinical condition and therefore improve their clinical management [18]. Moreover, it is designed to be used by every NICU professional with enough experience in the neonatal ward [18]. Since the rationale underlying the NAME model was described elsewhere (see [18] for more details of the NAME model significance), the present paper aims to begin the NAME validation process, investigating the model’s face, content and, as a preliminary analysis, construct validity.

## Material and methods

### The NAME model

The NAME model is a touch-based manual examination that produces two scores: 1) categorical, that includes three levels: “Bad,” “Marginal,” and “Good” – and 2) numerical – a 1-to-9 Likert scale (Table 1). The estimated time to perform the NAME is about 90 s [18].

**Table 1:** The NAME model score

Categorical score	Numerical score	Outcomes related to touch examination
Bad	1–3	Both compliance and homogeneity are absent
Marginal	4–6	Either compliance or homogeneity is present
Good	7–9	Both compliance and homogeneity are present

The operator evaluates how the infant reacts to light mechanical stimuli – pressure and distension – applied to the cranial and the sacral region of the body. The operator assesses two parameters: a) the compliance, that is whether the body changes its volume accordingly to the mechanical stimuli applied, and b) the homogeneity, that is whether the infant’s tissues adapt to the mechanical stimuli in the same way throughout the body [18]. The operator then gives the NAME score (Table 1).

The NAME construct can be summarised as follows (for a more detailed description, see Manzotti, Cerritelli, Chiera et al. 2020) [18]. To cope with external stimuli and stressors, infants might produce a neuromotor response through the activation of the autonomic nervous system (ANS) [19–21]. In particular, the ANS can elicit changes in the cardiovascular and respiratory systems, e.g., changes in heart rate, partial blood oxygen saturation monitored by pulse oximetry (SpO_2_), and breathing pattern [21–23].

Light touch, whether static or gentle, can induce peripheral and central physiological perturbations due to the stimulation of Merkel-neurite complexes and C-tactile fibers [24, 25]: the former release neurotransmitters that can alter the hemodynamics and smooth musculature activity, including respiration [26–29], whereas the latter can influence the central interoceptive system used by the organism to orchestrate the stress response and the ANS, thus altering the heart rate and breathing pattern [20, 30, 31].

When blood flow and breathing pattern change, body volume – how bodily fluids distribute in the various body segments and tissues – can change accordingly: therefore, light touch might induce a variation in body volume. The changes in the body volume and in the hemodynamic indexes depend on the ANS state of development, especially in infants: indeed, due to incomplete ANS development, preterm newborns could show disrupted cardiorespiratory adaptation and a different body volume change compared to full-term newborns. Similar cardiorespiratory and volume effects can be shown in infants with adverse clinical conditions as compared to healthy infants [23, 32, 33].

Changes in body volume can be felt by an operator through haptic perception – the process of perceiving an object features through touch. In particular, through the high sensitivity shown by the hands and fingertips, the operator can recognize changes in body volume as changes in the infant’s tissues softness [34–36]. The softness of an object depends on the object’s compliance – its ability to deform –, the amount of contact area between the hands and the tissues, and the distance a fingertip penetrates the tissue [37]. Based on the tissues changes felt through the hands, the operator can assess:

- compliance: whether the infant’s body as a whole can adapt accordingly to the manual stimuli, or poses some resistance;
- homogeneity: whether the adaptation is homogeneous throughout the body, or there are body areas that show different levels of softness or altered tissue responses – regions of interest (ROIs) that could reveal important information about infant’s conditions.

### NAME model validity

The main facets of validity can be resumed in the following questions: is the measure clear to be used? (face validity); Is the measurement scale relevant and exhaustive in relation to its construct? (content validity); Does the measure correlate with its underlying construct? (construct validity); Does the tool correlate with other established and valid instruments? (criterion validity) [1, 2].

To assess these types of validity (except criterion validity), the NAME model was discussed and examined by a team of NICU professionals, all with several years of experience in the paediatric/neonatology field and in the treatment of preterm newborns and working at the tertiary level NICU of “Vittore Buzzi” Children Hospital of Milan, Italy.

#### Face validity

Face validity refers to whether a measurement scale looks reasonable and it responds to the question whether the measurement has a clear meaning. Face validity involves a subjective judgement, and it is usually assessed by a panel of observers including experts, participants or researchers [2].

#### Content validity

Content validity refers to the degree to which a measuring tool reflects the construct that is being measured [1], and a careful analysis of content validity in the initial stages is encouraged to ensure the tool’s validity [2]. Content and face validity are two closely related types of validity and can be considered the minimum requirements for the acceptance of an index [2]. As for face validity, an expert panel is usually consulted, together with literature research about the construct that is going to be measured [1, 2].

However, content validity can also be assessed quantitatively through the content validity index (CVI), which measures the proportion of judges who agree on the relevance of the tool under investigation. The CVI can be measured both for every item (I-CVI) and for the whole scale (S-CVI) [38].

#### Construct validity

Construct validity is the degree to which a measuring tool correlates with the construct under investigation [1, 2]. It is the main form of test validation, using an indirect approach based on several measures [2]. Construct validity is strictly related to the theory underlying the test and to the hypotheses that the theory allows to make.

Since it involves hypotheses and correlations, construct validity can be measured quantitatively through statistical analysis [1, 2]: in particular, to evaluate construct validity, we tested hypotheses that stem directly from the rationale [18]. The following hypotheses for both the categorical and numerical score of the NAME model were tested in the present study:

- the NAME discriminates between healthy and complicated infants;
- preterm newborns have a lower NAME score than full-term ones: in other terms, prematurity shows an inverse correlation with NAME score;
- lower weight at birth correlates with lower NAME score;
- the age at the time of the NAME assessment correlates positively with the NAME score;
- the weight at the time of the NAME assessment correlates positively with the NAME score.

### Statistical analysis

#### Content validity

To assess the relevance of the tool’s items, the judges have to rate every item on a 4-point Likert scale, rating it as (1) “not relevant,” (2) “somewhat relevant,” (3) “quite relevant” or (4) “highly relevant” to the construct underlying the tool. The more an item is judged to be quite or highly relevant, the more it has content validity. The number of positive ratings (quite or highly relevant) is divided by the total number of ratings to obtain the I-CVI. The I-CVI can range from 0 to 1 and: higher than 0.80, the item is appropriate; lower than 0.70, the item must be eliminated; between 0.70 and 0.80, the item needs revision [38]. To account for chance agreement, for every I-CVI a modified kappa is calculated according to the formulae [39]:

1 $$ \mathrm{k}=\left(\mathrm{I}-\mathrm{CVI}-{\mathrm{p}}_{\mathrm{C}}\right)/\left(1-{\mathrm{p}}_{\mathrm{C}}\right) $$

2 $$ {\mathrm{p}}_{\mathrm{C}}=\left\{\mathrm{N}!/\left[\mathrm{A}!\ast \left(\mathrm{N}-\mathrm{A}\right)!\right]\right\}\ast 0.{5}^{\mathrm{N}} $$

where p_C_ is the chance agreement, N the number of judges, and A the number of judges who gave a 3 or 4 rating. A kappa higher than 0.78 means the item is considered excellent [39].

The more the number of items showing content validity, the more the scale shows content validity. To assess S-CVI, we computed the average S-CVI (S-CVI/Ave) that can be obtained dividing the sum of the I-CVIs by the number of items. S-CVI can also be computed as the universal agreement (S-CVI/UA), but S-CVI/UA does not account for chance agreement and it seems an overly conservative method if a judge misunderstands the underlying construct. An acceptable S-CVI/Ave should score higher than 0.90 [39, 40].

Therefore, to investigate content validity for the NAME model, we evaluated whether the two items (compliance and homogeneity) were considered relevant – through the CVI – and whether they can fully represent how the infant’s body tissues mechanically adapt to external stressors – through the expert panel.

#### Construct validity

Data were obtained from the evaluations made on 50 newborns hospitalized in the NICU by an expert operator (Male, 52y) with more than 10,000 h of clinical practice [41, 42], specific training in the pediatric field and more than 15 years of experience in the treatment of newborns. We collected information about the infants’ age and weight both at birth and at the time of the NAME assessment, and we calculated a “complexity index” to discriminate easily between healthy and complicated newborns. This index involved assessing the presence of complications in 10 health domains: intrauterine growth restriction, respiratory diseases, cardiovascular pathologies, gastrointestinal pathologies, urogenital diseases, neurological pathologies, metabolic alterations, genetic alterations, surgeries, and other problems (i.e., rare diseases). For every domain with one or more complications, the complexity index was increased by 1: therefore, it could range from 0 (no complications) to 10 (complications in every domain).

We described the general characteristics of the obtained sample, using mean (SD): gestational age, birth weight, age at the time of assessment, weight at the time of assessment. We then described the characteristics stratified by the NAME categorical score, reporting the number of Bad, Marginal, and Good newborns.

To assess the correlation between these five variables and the NAME score, we calculated Kendall’s τ correlations for the numerical score and both Kendall’s τ correlations and ordinal logistic regressions for the categorical score.

Data were analyzed using the free software R (Version 3.6.1, The R Foundation for Statistical Computing). Statistical significance was set for an alpha level of less than 0.05.

## Results

### Face validity

The panel of experts was composed by NICU professionals of “Vittore Buzzi” Children Hospital, including two neonatologists, a nurse, a physiotherapist, a psychologist and five osteopaths with years of experience in the paediatric field. A structured face validity process was established to consider the NAME model, from both a numerical and categorical standpoint, to be a useful index for improving communication among operators. The panel found the NAME useful for structuring codified procedures, for improving the clinical management of preterm newborns and for improving communication among operators. Therefore, we could conclude the NAME showed face validity for NICU professionals.

### Content validity

About content validity, the same expert panel judged both the NAME items to show high content validity; hence, the NAME scale showed good content validity (Table 2).

**Table 2:** Content validity index for the NAME items (I-CVI, k) and the whole scale (S-CVI/Ave)

Item	Number of 3 or 4 ratings out of 10 judgments	I-CVI^**a**^	p_**c**_	k
Compliance	10	1.00	0.001	1
Homogeneity	9	0.9	0.010	0.90
	**S-CVI/Ave**^**a**^	0.95		

*I-CVI* item content validity index; p_c_: change agreement, *S-CVI/Ave* average scale content validity index.

^a^ Calculations done according to Polit et al. 2007 [39]

The expert panel viewed compliance and homogeneity as not exhaustive of the infant’s general condition: many more physiological variables need to be measured to assess the infant’s clinical conditions. However, the panel agreed that the two items measure two different and valuable facets of the infant’s body response to external stimuli – how the whole body mechanically adapts to external stressors – and constitute what a touch-based assessment can evaluate about the behaviour of infants, especially if preterm.

### Construct validity

The 50 infants recruited were assessed and judged as Bad (*n* = 19), Marginal (*n* = 27), and Good (*n* = 4). Table 3 showed the characteristics of the sample. Both the Kendall’s τ correlations and the ordinal logistic regressions used for the NAME categorical score showed that infants who have higher age and weight, both at birth and at the time of the assessment, have more likelihood of receiving a good NAME categorical score. Instead, a greater complexity index decreased the infant’s likelihood to show a good body adaptation (Table 4).

**Table 3:** General characteristics of the sample divided by NAME categorical score

	Total	Bad	Marginal	Good
*N*	50 (100%)	19 (38%)	27 (54%)	4 (8%)
Gestational age (wks)	34.8 (3.9)	33.2 (4.1)	35.3 (3.3)	39.5 (1.3)
Birth weight (g)	2181.2 (882.5)	1678.5 (804.7)	2421.7 (802.1)	2946.2 (598)
Post-menstrual age at assessment (wks)	37.8 (3.3)	36.8 (3.3)	37.9 (3.0)	41.5 (2.4)
Weight at assessment (g)	2468.9 (793.9)	2130 (734.4)	2612.6 (754.5)	3108.8 (815.5)
Complexity index	2.1 (1.2)	2.6 (1.2)	1.8 (1.0)	2.0 (1.4)

Values shown are *N* (%) and mean (standard deviation).

**Table 4:** Preliminary results for the NAME categorical score (Kendall’s τ correlation and ordinal logistic regression)

	Kendall’s τ (CI 95%)	***p***-value	OR (CI 95%)	***p***-value
Gestational age^a^	0.38 (0.20, 0.53)	0.002	1.30 (1.10, 1.57)	0.004
Birth weight^b^	0.45 (0.28, 0.59)	< 0.001	1.14 (1.06, 1.24)	0.001
Post-menstrual age at assessment^a^	0.28 (0.10, 0.45)	0.018	1.24 (1.04, 1.52)	0.023
Weight at assessment^b^	0.31 (0.13, 0.47)	0.006	1.11 (1.03, 1.21)	0.010
Complexity index^c^	−0.31 (− 0.47, − 0.12)	0.016	0.56 (0.32, 0.94)	0.034

^a^ Gestational age and age at assessment were measured in weeks

^b^ Birth weight and weight at assessment were measured in hectograms

^c^ Complexity index was calculated as the number of complications (e.g., surgery, intrauterine growth restriction) and organs/systems affected by pathologies

The Kendall’s τ correlations showed the same results for the NAME numerical score. Gestational age and weight, both at birth and at the time of the assessment, correlated positively with the NAME numerical score. Instead, the complexity index correlated negatively with the NAME numerical score: more complicated babies showed worse body adaptation (Table 5).

**Table 5:** Preliminary results for the NAME numerical score obtained through Kendall’s τ correlation

	Kendall’s τ (CI 95%)	***p***-value
Gestational age^a^	0.35 (0.17, 0.50)	0.001
Birth weight^b^	0.41 (0.24, 0.56)	< 0.001
Post-menstrual age at assessment^a^	0.29 (0.10, 0.45)	0.008
Weight at assessment^b^	0.31 (0.13, 0.48)	0.003
Complexity index^c^	−0.32 (− 0.48, − 0.14)	0.005

^a^ Gestational age and age at assessment were measured in weeks

^b^ Birth weight and weight at assessment were measured in hectograms

^c^ Complexity index was calculated as the number of complications (e.g., surgery, intrauterine growth restriction) and organs/systems affected by pathologies

## Discussion

This study investigated the NAME model face, content, and, as preliminary analysis, construct validity. Results demonstrate that the NAME model seems to have good validity. The expert panel gathered to assess face validity viewed the two items of compliance and homogeneity as highly relevant to evaluate infants and to communicate about infants in the neonatal ward. The same expert panel judged the NAME model also to have excellent content validity measured through the S-CVI/Ave [39].

However, a diagnostic tool can be considered valid only if it shows construct validity, which directly connects to the theory [1]. Using a representative sample of hospitalized babies, we found that both gestational age and bodyweight correlated positively with the NAME score, whether categorical or numerical. In particular, we found that both gestational age and birth weight correlated stronger with the NAME score than their counterparts at the time of assessment. From a clinical standpoint, this result is relevant as both low gestational age and low birth weight represent severe risk factors for the infant’s growth and neurological development [6, 43, 44].

The complexity index we calculated to differentiate healthy and complicated babies correlated negatively with the NAME score – the more the pathologies, the less the capacity of the baby’s body to adapt to an external stressor. This finding supports our hypothesis that the NAME model can discriminate between healthy and complicated infants. The two scores, categorical and numerical, seemed to behave similarly, even though, at first, the numerical scale could be viewed as more sensitive due to being larger than the categorical scale. This result supports the hypothesis that the categorical scale made of the scores Bad, Marginal, and Good can efficiently categorize the infants according to their conditions. Therefore, the NAME could become part of the neonatology ward routine-care to assess the infant’s general clinical condition.

Regarding other widely used procedures, such as the Alberta Infant Motor Scale [15] or the Assessment of Preterm Infants’ Behavior [14], the NAME can be performed in the crib/incubator, even when the infant is asleep, thus reducing the risk of distressful position or maneuver. For its intrinsic easiness, the NAME can also be applied in partially stable infants. Based on the literature, it took less time to be performed compared to other procedures. Except for pain assessment [45], those procedures take from 30 min – for assessing newborns, to several hours – for writing the clinical report [14, 46]. Concerning a previous attempt [16] to codify a neonatal manual procedure, the NAME is more straightforward since it involves static or gentle touch that can be performed by every professional using touch-based procedures. Moreover, it is far less stressful since the previous proposed assessment procedure lasted 10 min.

Future research should further test the NAME model to make sure it could be efficiently introduced in clinical practice and help professionals to define good therapeutic plans – in particular, its advantages over the existing assessment procedures need to be consistently and specifically tested. A new test should indeed be easier to use, take less time to be performed, show better predictive capacity, or reduce the costs [1].

Content validity might require an additional study. One key question we might pose is: Does the measurement scale include every aspect related to the underlying construct – the rationale – and exclude what is irrelevant? [2] At this stage, it is difficult to answer in a definitive way to such a question, especially in the medical field, where researchers and professionals deal with complex systems [2]. However, future studies might shed light on additional elements to take into account. Following the expert panel’s judgment, the NAME model might also be enhanced by integrating physiological parameters like heart rate, respiratory rate, blood pressure, SpO_2_, temperature (vital signs already monitored in NICUs) [47, 48], or heart rate variability – a measurement that is emerging as reliable and valid to predict and monitor the infants’ clinical progression [49].

In the same way, the assessment of construct validity requires further evidence. Despite the positive results, the significant correlations between the NAME score and the infants’ characteristics (age, weight, and complexity index) were not perfect – the Kendall’s τs were far from the score of 1 (perfect agreement). This result was expectable: weight and gestational age are just two characteristics that may give important information about the babies’ health, but they certainly do not depict the whole clinical picture. Concerning the complexity index, a limitation is that it was calculated evaluating the quantity, not the severity of complications: in fact, it is conceivable that infants with fewer but more severe complications might receive a worse score than infants with more complications that are, however, less severe. Indeed, the present paper represents just the first step of the NAME validation process: further studies should assess the correlation between the NAME score and specific clinical outcomes, and even whether changes in the NAME score could recognize acute changes in clinical conditions, e.g., in case of sepsis or other emergencies. Besides, could ROIs on the infant’s body indeed be found? And could they correlate with specific clinical conditions? (e.g., could the ROIs in the upper thorax correlate with respiratory pathologies?).

In the present paper, we assessed only discriminant validity, which is whether the test can discriminate among people with different conditions (e.g., healthy vs. diseased) [1]. Still, other forms of construct validity can be assessed. Examples are convergent validity – if the test correlates with the measurements of other instruments that can assess some elements of the underlying construct – and divergent validity – if the test fails to correlate with tools that relate to different constructs [1].

We could not assess criterion validity, the fourth facet of validity, due to a lack of a gold standard test that evaluates how the infant’s body mechanically adapts to external stressors. Indeed, criterion validity compares the new test being assessed and an existing criterion that is established as valid, and it can be evaluated as concurrent validity and predictive validity. Concurrent validity is assessed when the new test is compared in the present with an existing criterion that measures the same construct. Predictive validity is assessed when the new test is compared with a criterion in the future. When possible, the criterion used for comparison should be a gold standard [1, 2]. Future studies could evaluate criterion validity through correlating the NAME score with other scales that measure the infant’s development or conditions.

## Conclusions and future perspectives

The neonatology field is changing at a high-speed rate to improve health care assistance. This challenge is also tackled by the growing interest and need for developing reliable instruments that can assess newborns and help NICU professionals to improve care. As preliminary results, the NAME appeared to be a tool well accepted by NICU professionals and relevant for evaluating the infant’s response to external stressors, i.e., touch. It showed initial construct validity, thus supporting its usefulness in recognizing and discriminating the infant’s ability to adapt to external stressors and the infant’s general condition. However, assessing validity is a long process since it needs to use a variety of approaches and to test several hypotheses related to the underlying construct [2]. Therefore, further studies are needed to complete the validation process of the NAME.

These future studies must test the NAME model predictive capacity to answer the question: can the score correlate with or predict the infant’s clinical conditions, or at least his developmental trajectory? Since the changes in body volume assessed by the NAME procedure depend on the infant’s ANS development, the NAME could give information about the infant’s conditions and development [23, 32, 33]. If future evidence supports this hypothesis, it will become paramount to compare the NAME model with other assessment procedures with good predictive capacity.

## Data Availability

All data generated or analysed during this study are included in this published article.

## Abbreviations

ANS: Autonomic nervous system
CVI: Content validity index
I-CVI: Item content validity index
NAME: Neonatal assessment manual score
NICU: Neonatal intensive care unit
ROI: Region of interest
S-CVI: Scale content validity index
S-CVI/Ave: Average scale content validity index
S-CVI/UA: Universal agreement scale content validity index
SpO_2_: Partial blood oxygen saturation monitored
